# Comparative pharmacokinetic evaluation of nanoparticle-based vs. conventional pharmaceuticals containing statins in attenuating dyslipidaemia

**DOI:** 10.1007/s00210-024-03140-5

**Published:** 2024-05-08

**Authors:** Jacob Cordina, Isha Ahmad, Rohan Nath, Bahara Abdul Rahim, Andrew Van, Dalya Al-Zuhairi, Kylie Williams, Lisa Pont, Rachelle Catanzariti, Samir Mehndiratta, Rayen Yanara Valdivia-Olivares, Gabriele De Rubis, Kamal Dua

**Affiliations:** 1 Discipline of Pharmacy, Graduate School of Health, University of Technology Sydney, Ultimo, NSW 2007 Australia; 2 Faculty of Health, Australian Research Centre in Complementary and Integrative Medicine, University of Technology Sydney, Ultimo, NSW 2007 Australia; 3 Departamento de Farmacia, Escuela de Química y Farmacia, Facultad de Química y de Farmacia, Pontificia Universidad Católica de Chile, 7820436 Santiago, Chile; 4 Institute for Biological and Medical Engineering, Schools of Engineering, Medicine and Biological Sciences, Pontificia Universidad Católica de Chile, 7820436 Santiago, Chile

**Keywords:** Pharmacokinetics, Statin, Statin nanoparticles, Drug delivery, Dyslipidaemia

## Abstract

Dyslipidaemia describes the condition of abnormal lipid levels in a person’s bloodstream. Since the 1980s, statin medications have been used to treat dyslipidaemia and other comorbidities, such as stroke risk and atherosclerosis. Statin medications were initially synthesised from fungal metabolites, but many synthetic statin drugs have been manufactured since then. Statin medication is quite effective in reducing total cholesterol levels in the bloodstream, but it has limitations. Due to their poor water solubility, statin drugs possess poor oral bioavailability, which hinders their therapeutic efficacy. Nanoparticle drug delivery technology has been shown to improve the pharmacokinetic profiles of many drug classes, and statins have great potential to benefit from this. This paper reviewed the currently available literature on nanoparticle statin medication and evaluated the possible improvements that can be made to the pharmacokinetic profile and efficacy of conventional statin medication. It was found that the oral bioavailability of nanoparticle medication consistently outperformed conventional medication by up to 400% in some cases. Substantial improvements in time to peak plasma concentration and plasma concentration peaks were also found, and increased periods in circulation before excretion were shown. It was concluded that nanoparticle technology has the potential to completely replace conventional statin medication as it offers more significant benefits with minimal drawbacks. Upon further study and development, the manufacture of nanoparticle statin medication should become feasible enough for large-scale application, which will significantly benefit patients and unburden healthcare systems.

## Introduction

The recent development of nanoparticle technology as an innovative method for delivering pharmaceuticals shows excellent potential, generating significant attention within the medical and pharmaceutical industries. Nanoparticle drug delivery systems possess the potential to significantly transform treatment in multiple domains, encompassing efficacy, stability, absorption, shelf-life, and augmented carrier capacity. This study aims to assess the present state and prospective uses of nanoparticle-based statin medicines, a vital category of pharmaceuticals involved in lipid regulation. This evaluation entails a direct comparison with traditional statin medicines commonly used on the market. The primary objective of this study is to thoroughly investigate the overall pharmacokinetic characteristics of nanoparticle-based statins compared to conventional statins. Furthermore, the study examines the impact of these drugs on patient outcomes and investigates any limits that may be linked to their utilisation.

High levels of cholesterol increase the risk of stroke and heart disease. It contributes to one-third of global ischemic heart disease cases. According to the World Health Organization, in 2008, 39% of the population suffered from dyslipidaemia, with 40% of females and 37% of males affected (Australian Bureau of Statistics 2013; Institute and of Health and Welfare 2017). The risk of dyslipidaemia increases with age. According to the Australian Health Survey 2013, dyslipidaemia prevalence doubled from the age category of 45–54 to 55–65 from 7 to 14%, and it increased to 21% for the age of 65 and above (Australian Bureau of Statistics 2013; Institute and of Health and Welfare 2017).

Dyslipidaemia is a risk factor that can be modified and is strongly associated with various diseases such as atherosclerotic cardiovascular diseases (Jean et al. 2015) and cancer (Schachter 2005). Dyslipidaemia is defined as having a total blood cholesterol level equal to or greater than 5.5 mmol/L (Institute and of Health and Welfare 2017). It is characterised by abnormal plasma lipid levels, including low-density lipoprotein cholesterol (LDL-C), high-density lipoprotein cholesterol (HDL-C), and triglycerides in the circulatory system. It has been the subject of substantial investigation in many published research investigations. The Heart Foundation Australia has employed statin medicine as the principal pharmacological intervention for individuals with increased cholesterol levels.

Statins, which are recognised as inhibitors of 3-hydroxy-3-methylglutaryl-coenzyme A (HMG-CoA) reductase, constitute a well-acknowledged pharmaceutical category that exhibits advantageous therapeutic properties in the treatment of cardiovascular disease and lipid disorders. Furthermore, these drugs possess prospective applications in the field of cancer treatment. Nevertheless, the bioavailability of statins is hindered due to their limited solubility in water and fast metabolic breakdown (Montelione et al. 2023; Navya et al. 2019).

Statin drugs face issues regarding their limited bioavailability (Arca and Pigna 2011; Montelione et al. 2023; Schachter 2005), which nanoparticle-based therapy may address (Montelione et al. 2023). In recent studies, researchers have explored the utilisation of polymers and nanoparticles to enhance the bioavailability and efficacy of statin delivery. The utilisation of polymers and nanoparticles has been shown to facilitate the delivery of statins and enhance their therapeutic effects (Ahmed et al. 2015; De Jong 2008; Jose 2016; Raza et al. 2017; Sever et al. 2003; Shepherd et al. 1995; Shepherd et al. 2002; Shilpi et al. 2017; Matsumoto et al. 2022). This is achieved through enhancing oral bioavailability and promoting target-specific interactions. Consequently, these interventions reduce vascular endothelial dysfunction, intimal hyperplasia, and ischemia–reperfusion injury and promote cardiac regeneration. Additionally, they facilitate positive remodelling in the extracellular matrix, reduce neointimal growth, and enhance re-endothelization*.*

The utilisation of nanotechnology has resulted in the development of an improved system that exhibits increased water solubility, absorption, bioavailability, efficacy at lower doses, and controlled drug release at the site of administration (Jose 2016*).* The emerging utilisation of nanoparticle technology as a novel approach to medicinal delivery has gained considerable interest and attention in the medical and pharmaceutical sectors. Nanoparticle drug delivery systems can revolutionise treatment, including efficacy, stability, absorption, shelf-life, and enhanced carrier capacity (Jose 2016; Raza et al. 2017). This study directly compares nanoparticle statins with conventional statin medications that are widely used on the market. The main aim of this study is to comprehensively examine the pharmacokinetic properties of nanoparticle-based statins compared to conventional statins.

The scope of this study is restricted to attenuating dyslipidaemia and will not focus on managing atherosclerosis and cardiovascular risk mitigation. This review will analyse the current literature regarding the pharmacokinetic profiles of conventional and nanoparticle statin medications. It will also illustrate the efficacy of both classes and synthesise a generalised conclusion regarding whether the new nanoparticle technology surpasses the presently established conventional statin medication.

## Pharmacokinetic profile of conventional statin medication

Statins display varied pharmacokinetic characteristics, leading to distinct clinical implications regarding safety and potential drug interactions. Several authors have presented comparative pharmacokinetic data for the various statins currently available for clinical use, and these can be seen in Table 1.

**Table 1:** Comparative pharmacokinetic properties of statins (Corsini et al. 1999)

Type of statin	Absorption %	Bioavailability	Metabolism	Effect of food on bioavailability	Solubility	Protein binding %	Half-life (hr)
Atorvastatin	30	12	CYP3A4	Bioavailability decreased	Lipophilic	98	14
Fluvastatin	98	19–29	CYP2C9	Bioavailability decreased	Lipophilic	> 98	4.7
Lovastatin	30	5	CYP3A4	Bioavailability increased	Lipophilic	95	2
Cerivastatin	98	60	3A4, 2C8	No effect	Lipophilic	> 99	2.5
Simvastatin	60–80	< 5	CYP3A4	No effect	Lipophilic	95–98	2
Rosuvastatin	N/A	20	CYP2C9 (minor)	No effect	Hydrophilic	90	19
Pravastatin	34	18	Sulfation	Bioavailability decreased	Hydrophilic	50	1–2

## Absorption

There is a vast difference between the absorption rates of HMG-CoA reductase inhibitors, starting from 30% for drugs like atorvastatin and lovastatin to as high as 98% for cerivastatin and fluvastatin (Table 1) (Corsini et al. 1999). Food intake is a factor that can affect the bioavailability of the drug. While atorvastatin and fluvastatin see reduced bioavailability with food intake, simvastatin and rosuvastatin remain unaffected (Table 1). Apart from cerivastatin, all statins have limited systemic bioavailability, indicating they undergo significant first-pass metabolism; this is a limitation of orally administered statins. The current statin-class medications on the market possess characteristically low bioavailability. This is generally a result of substantial first-pass extraction and poor water solubility. Atorvastatin, rosuvastatin, and simvastatin display bioavailability levels of 12%, 20%, and 5%, respectively.

## Distribution

A drug is generally not pharmacologically active when bound to plasma proteins. The unbound fraction is the one that can exert a therapeutic effect and can be metabolised and excreted (Randomised trial of cholesterol lowering in 4444 patients with coronary heart disease: the Scandinavian Simvastatin Survival Study (4S) 1994). Except for pravastatin, all statins have a high affinity for plasma proteins (Table 1). Consequently, the concentration of the free, active form of the drug in the system is comparatively low. The hydrophilic properties of pravastatin limit its extensive distribution in tissues, even though the levels of unbound pravastatin in circulation are higher than the other statins (Farjadian et al. 2019). Statins vary in lipophilicity; rosuvastatin and pravastatin are hydrophilic, while the others are lipophilic, as seen in Table 1. While both groups inhibit HMG-CoA reductase, they do so via distinct pathways. Lipophilic statins can passively permeate the liver cell membrane, whereas hydrophilic statins need carrier-mediated transport for uptake into the liver (Brault et al. 2014).

## Metabolism

Statins are primarily broken down by the cytochrome P450 (CYP450) group of enzymes, encompassing more than 30 distinct isoenzymes (Patra et al. 2018). The CYP3A4 isoenzyme metabolises atorvastatin, lovastatin, simvastatin, and cerivastatin (also metabolised by CYP2C8). The CYP2C9 isoenzyme metabolises fluvastatin and partially metabolises rosuvastatin (Table 1).

## Excretion

For most statins, the elimination route is through the bile after being metabolised by the liver. The half-life (*t*_1/2_) represents the duration required to decrease the drug’s concentration in the system by half and indicates the overall rate of drug clearance. Fluvastatin, lovastatin, pravastatin, and simvastatin possess a comparatively short half-life, making them ideal for evening administration or, in the cases of fluvastatin and lovastatin, for use in extended-release forms to enhance their efficacy. Conversely, atorvastatin, pitavastatin, and rosuvastatin have more extended half-lives, allowing for flexible dosing at any time during the day (Patra et al. 2018; Sizar et al. 2023).

## Pharmacokinetic profile of nanoparticle statin medication

**Fig. 1:**
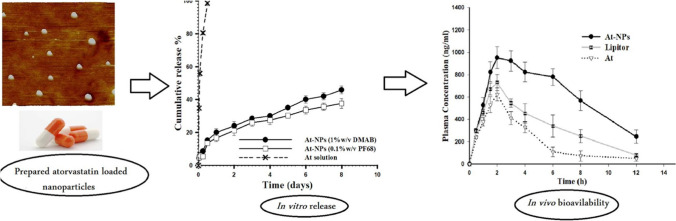
Enhancement of atorvastatin oral bioavailability via encapsulation in polymeric nanoparticles. Reproduced with permission (Shaker et al. 2021)

**Fig. 2:**
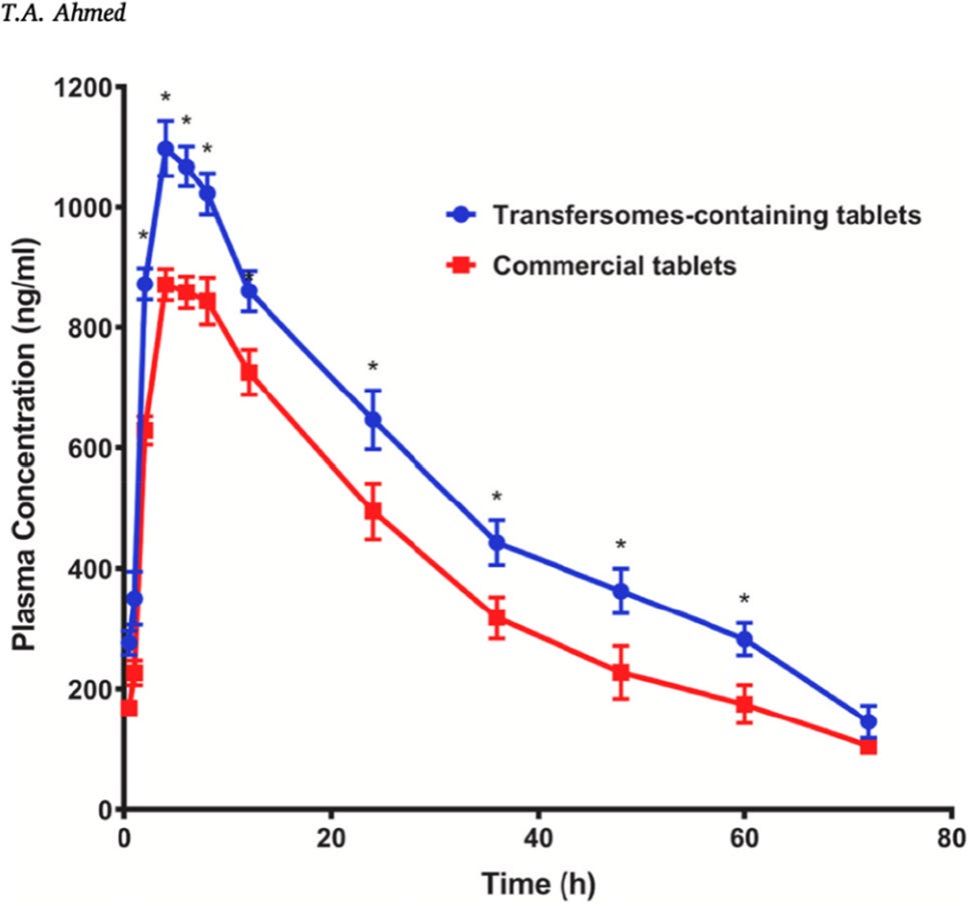
Graph of in vitro and in vivo results of conventional vs. NP rosuvastatin. Substantial improvement to bioavailability was demonstrated. It takes a similar time to release in the system, but substantially higher plasma concentrations are achieved for a prolonged period. Reproduced with permission (Ahmed 2021). *: *P*<0.05 between the two groups

Conventional statins have low cell permeability due to low water permeability. When covered by water-soluble carriers, e.g. liposomes, this will increase water solubility and oral bioavailability of statins, thus increasing circulation time and the retention effect. The statin carrier facilitates receptor access and cell membrane crossing by adsorptive endocytosis, increasing statin bioavailability (Figs. 1 and 2) and effectiveness. The pharmacokinetic properties of statin nanoparticles can differ with different types of statins, as all statins have the exact mechanism of action but different physicochemical properties (e.g. water solubility, chemical structure, and pharmacokinetic properties).

## Mechanism of action of nanoparticle statins

Statin medications reduce cholesterol biosynthesis mainly in the liver and modulate lipid metabolism. This is carried out by the inhibition of HMG-CoA reductase. As mentioned earlier, statin efficacy is challenged by poor oral absorption, low bioavailability, and low water solubility. Consequently, to maintain therapeutic efficacy and overcome these pharmacokinetic barriers, high doses are often required. High-dose statin prescribing is common, and logically, it follows that increasing doses of statin medication is correlated with the increased likelihood of dose-dependent adverse effects and drug intolerance (Brault et al. 2014; Climent et al. 2021; Long-Term Intervention with Pravastatin in Ischaemic Disease (LIPID) Study Group 1998; Etemad et al. 2023). Therefore, the utilisation of nanocarriers for statin delivery is aimed at circumventing these conventional barriers allowing for the natural mechanism of statin medication to be carried out efficaciously at lower doses and thus reducing adverse drug reactions and consolidation of medication resources. There are many possible ways in which nanocarriers may overcome these challenges due to the inherent dynamic possibilities nanocarriers offer. Nanoparticles coated in caseinate can reduce water solubility (Ahmed et al. 2015), statin encased liposomes can increase oral bioavailability (Etemad et al. 2023), and even direct nanocarrier drug delivery to the desired area (Matsumoto et al. 2022) may be utilised to improve the current conventional limitations.

## Absorption of nanoparticles

Oral absorption of nanoparticles in drug delivery systems depends on particle size, surface chemistry, and formulations. The size of a particle impacts its solubility, bioavailability, drug loading, release, cellular distribution and cell accumulation, drug stability, and overall drug effectiveness. Particle sizes smaller than 40 nm have lower cellular uptake than larger particles, e.g. 50 nm (Jean et al. 2015). The optimal accumulation and circulation are achieved with particle sizes between 100 and 200 nm (Long-Term Intervention with Pravastatin in Ischaemic Disease (LIPID) Study Group 1998).

Surface charge is another factor that influences particle interaction and cellular entry. A negatively charged particle has a higher uptake in specific cells, e.g. tumour cells, and lower uptake in liver cells, which helps reduce metabolic degradation and clearance of the drug by the liver. Negatively charged particles are best for target drug delivery. Negatively charged particles are also more stable and prevent particle aggregation in the biological environment.

A positively charged particle can easily penetrate the cell membrane due to electrostatic attraction between positively charged nanoparticles and the negatively charged cell membrane. It is used for nucleic acid delivery into the cell, which can easily cross the negatively charged cell membrane. However, there is increased toxicity related to positively charged particles compared to negatively charged particles (Jean et al. 2015).

The surface chemistry design of nanoparticles can play a significant role in cellular uptake and stability and reduce toxicity. This can increase blood circulation time and therapeutic effects, e.g. gold nanoparticles in cancer treatment.

## Bioavailability

Due to poor water solubility, conventional statin medications suffer from poor oral bioavailability. It is this pharmacokinetic property in which nanoparticle technology has the most significant opportunity for improving patient outcomes and dyslipidaemia attenuation (Table 2). There is substantial variance in mechanism between the diverse range of nanoparticle technology, and thus, specific nanoparticle statin medications may have different pharmacokinetic properties than other nanoparticle medications. However, what is possible is to synthesise a consensus and produce a generalised pharmacokinetic profile of all statin-class nanoparticle medications (Ahmed 2021; Arca and Pigna 2011; Australian Bureau of Statistics 2013; Institute and of Health and Welfare 2017; Corsini et al. 1999).

**Table 2:** Improvements to bioavailability found in research (Ahmed et al. 2015; Arca and Pigna 2011; Montelione et al. 2023; Shaker et al. 2021; Shilpi et al. 2017)

Study type	Comparative factor	Results	Reference
In vitro and in vivo data	Concentration over time of conventional rosuvastatin tablet medication and lyophilized orodispersible tablets containing transferosomes nanoparticles	134% increase to oral bioavailability	Ahmed 2021)
In vitro and in vivo data	Oral bioavailability of conventional atorvastatin medication and cellulose-free polymers, compounds with poly-oxy-propylene (hydrophobic fraction), and poly-oxy-ethylene (hydrophilic fraction)	400% increase to oral bioavailability	Montelione et al. 2023)
In vitro and in vivo data	Oral bioavailability of conventional atorvastatin and atorvastatin containing polymeric nanoparticle medication	385% increase to oral bioavailability	Shaker et al. 2021)
In vitro and in vivo data	Oral bioavailability of conventional simvastatin and simvastatin-zein nanoparticles coated with caseinate	400% increase to bioavailability	Ahmed et al. 2015)
In vitro data	Oral bioavailability of conventional atorvastatin medication and loaded stearic acid modified gelatine nanoparticles	Improved oral bioavailability	Shilpi et al. 2017)

## Efficacy profile of conventional statin class medications (De Jong 2008; Jose 2016; Raza et al. 2017; Schachter 2005; Shaker et al. 2021; Shepherd et al. 1995)

Cardiovascular disease (CVD) ranks among the foremost global health concerns, responsible for a staggering one-third of worldwide fatalities, equating to 17.3 million deaths annually. The introduction of conventional statins represented a pivotal moment in achieving greater LDL reduction efficacy compared to earlier approaches. Furthermore, research has supported cholesterol reduction’s advantages through statins in primary and secondary cardiovascular disease prevention. Meta-analyses, including clinical trials investigating statins’ cardiovascular effects, revealed consistent and proportional decreases in the risk of experiencing new significant vascular or coronary events, irrespective of patient’s age, gender, cholesterol levels, diabetes status, hypertension, previous heart attacks, or other coronary conditions.

Traditional statins are a standard class of medications prescribed to lower cholesterol levels. TGA-approved statins include atorvastatin, rosuvastatin, simvastatin, pravastatin, and fluvastatin. Notably, statins are part of a comprehensive strategy for managing cardiovascular risk, including incorporating lifestyle modifications. While they are generally well received, knowing they can carry potential side effects requires oversight during their administration and therapeutic duration. The FDA-approved uses may differ slightly among different statins but generally include the treatment and/or prevention of clinical atherosclerotic cardiovascular disease (ASCVD), both in terms of primary prevention (e.g. preventing the first occurrence) and secondary prevention (e.g. reducing the risk of recurrent events like myocardial infarction or stroke).

On a biochemical level, statins are selective, competitive HMG-CoA reductase inhibitors. Statins block the activity of an enzyme known as HMG-CoA reductase, which plays a crucial role in manufacturing cholesterol within the liver. This inhibition decreases the production of cholesterol and prompts a growth in the quantity of LDL receptors in liver cells. As a result, this reduces LDL cholesterol levels in the blood, reducing cardiovascular disease risk. Furthermore, statins may exert mild effects on triglycerides and HDL cholesterol levels (Montelione et al. 2023; Raza et al. 2017).

Several meta-analyses of randomised control and clinical trials worldwide have shown the benefits of statins in people with cardiovascular risk factors. Statins effectively prevent mortality and cardiovascular-related illness in individuals with a low risk of cardiovascular issues. The reductions in relative risk were comparable to those observed in patients who had previously experienced coronary artery disease. Furthermore, statin therapy for primary prevention has been reported to display a decreased likelihood of all-cause mortality and CVD events for adults at risk of CVD but have not encountered CVD events in the past. These advantages of statin therapy extend to a wide range of demographic and clinical groups, with consistent relative benefits found among various groups defined by their demographic and clinical characteristics. Even in individuals who do not have a known history of cardiovascular disease but possess risk factors for heart-related issues, the use of statins was linked to notably improved survival rates and substantial decreases in the likelihood of experiencing significant cardiovascular events in the future.

Most research studies and published journal articles consistently indicate minimal risk of adverse events associated with statins and do not overshadow their effectiveness in preventing cardiovascular disease. This suggests that, based on patient outcomes, the balance between statins’ benefits and potential harms is favourable. However, it is crucial to emphasise that these findings should not be used as definitive evidence for the universal use of statins. Instead, they should be considered supportive information to aid healthcare professionals in making informed clinical decisions tailored to individual patients.

## Efficacy profile of nanoparticle statin medication

The main effect of statins is to reduce LDL-C, with their efficacy in lowering LDL-C levels from 10 to 40% (Table 3) (Montelione et al. 2023). According to the literature available, enhancing the bioavailability of statins can potentially reduce the adverse effects and toxicity linked to higher levels of statins in the bloodstream. The utilisation of efficient delivery mechanisms can contribute to optimising this bioavailability.

**Table 3:** The impact of nanocarriers containing statins on the extracellular matrix (Montelione et al. 2023)

Drug	Carrier type	Diameter (nm)	Targeted cell types/receptor	Outcomes compared to the free drug	Superior to free drug?
Pitavastatin	PLGA	196	Alveolar macrophages, smooth muscle cells	eNOS ↑ (40%), NF-kB ↓(60%) smooth muscle cells ↓ pulmonary hypertension ↓ survival ↑ (20%) chemotactic proteins ↑ post-ischemic permeability ↑ grown factor ↑ micro/macrovascular angiogenesis ↑ monocyte mobilisation ↓	Yes
Simvastatin	PLGA	233	VCAM-1		No
Simvastatin	DSPC, DSPG, cholesterol	164	Macrophages, monocytes	Monocytes ↓ (24%), stenosis ↓ (33%)	Yes
Simvastatin	rHDL	26	Macrophages, endothelial cells	Plaque area ↓ (36%), macrophages ↓ (84%), inflammation ↓ fibrosis↓	Yes
Pravastatin	PDMS/PMOXA	97	SR-A1	LDL uptake ↓	Yes

Nanotechnology presents advantages in statin delivery, particularly concerning enhancing oral bioavailability. Exploring at least two processes is essential to enhance the oral bioavailability of statins. Two main factors might affect the effectiveness of statins in the body. First, enhancing their solubility in the gastrointestinal tract can lead to better absorption. Second, minimising or eliminating first-pass metabolism after oral intake can prevent certain statins from reaching the desired levels in the body. Furthermore, nanomedicine has developed into a developing area of research, demonstrating considerable efficacy in treating diverse medical conditions (Jose 2016). As indicated by its terminology, the field under consideration focuses on physiological phenomena occurring at the nanoscale, hence aiding in advancing the development of non-intrusive apparatus such as diagnostic tools, transport mechanisms, and other medically valuable instruments.

Considerable work has been dedicated to designing and developing an optimal delivery system that maximises the therapeutic substance’s safety, selectivity, bioavailability, and efficiency. The utilisation of nanotechnology facilitates the administration of the treatment. Upon delivery, nanoparticles are introduced into the bloodstream, where they avoid the reticuloendothelial system and disperse throughout the extracellular matrix. Moreover, nanoparticles can migrate towards specific cells inside the tissue and then gain entry through endocytosis. The medications undergo absorption and subsequent release. Based on scientific investigations, nanoparticles have been found to facilitate the safe and effective transportation of therapeutic substances to their intended destinations. Polymeric nanoparticles, cationic lipids, liposomes, dendrimers, and inorganic nanoparticles represent a range of nanomaterial-based delivery strategies that have gained significant prominence in various applications (Downs et al. 1998; Montelione et al. 2023).

The advantageous applications of these materials stem from their unique characteristics, such as their capacity to degrade naturally, compatibility with biological systems, reduced immune response, decreased toxicity with convenient modification options, and improved delivery of pharmaceuticals or genetic material for disease treatment. Numerous research studies have investigated the transfer of medications or genetic material to reduce plasma cholesterol levels and thus treat cardiovascular diseases. Using polymers and nanoparticles has been shown to enhance the delivery and efficacy of statins through mechanisms such as enhancing oral bioavailability and facilitating target-specific interactions. These advancements have demonstrated potential in reducing vascular endothelial dysfunction, intimal hyperplasia, and ischemia–reperfusion injury while also promoting cardiac regeneration, positive renovation in the extracellular matrix, decreased neointimal growth, and increased re-endothelialisation (Montelione et al. 2023).

## Anti-inflammatory effect

Statin nanoparticles aim to reduce inflammation in atherosclerosis. According to a research study by Duivenvoorden et al. (2014), HDL nanoparticles reduce atherosclerosis plaque inflammation by mevalonate pathway inhibition and decrease inflammatory monocytes. The study used pitavastatin nanoparticles to investigate the enhancement of the drug’s therapeutic efficacy, the preservation of the active molecule against enzymatic breakdown within tissues, and the fact that the active component experiences a decrease in renal excretion.

## Limitations of both classes

HMG-CoA reductase inhibitors, also called statins, are essential for treating dyslipidaemia. Statins based on nanoparticles have been proposed to enhance pharmacokinetics and therapeutic results in the pharmaceutical industry. However, both conventional and statin formulations based on nanoparticles have drawbacks.

Nanoparticle-based drug formulation frequently necessitates a complex, technologically advanced manufacturing process that can be more resource-intensive than conventional drug manufacturing (Jose 2016). Despite the nanoparticles’ protection ability, they may show instability under certain circumstances, which could shorten a drug’s shelf life. The potential for cellular damage or unintended immune reactions that nanoparticle-based drugs may elicit, signalling potential toxicity, is a significant concern.

Additionally, the complex manufacturing processes and requirements for specialised equipment may drive up the price of these medications, reducing their availability to many patients (De Jong 2008). Recent studies have highlighted nanoparticle statins’ improved absorption and bioavailability, attributing these advantages to their small size and increased surface area. The available literature does, however, also highlight variation in absorption across various nanoparticle formulations, suggesting a potential source of unpredictable pharmacokinetic results.

Conventional statins, on the other hand, come with their own set of problems. Due to hepatic first-pass metabolism, their oral administration frequently encounters difficulties that limit their bioavailability and, as a result, their therapeutic efficacy (Long-Term Intervention with Pravastatin in Ischaemic Disease (LIPID) Study Group 1998). Statins with well-known names like atorvastatin and simvastatin have corresponding muscle side effects and potential liver toxicity. These adverse effects may hamper the long-term use of some patient groups. The increased risk of drug interactions is another urgent issue, particularly when these statins are co-administered with medications like antifungals, antibiotics, or HIV (Raza et al. 2017). Additionally, some conventional statins’ inherent pharmacokinetic characteristics may require patients to take them more frequently, making it difficult for them to comply with treatment regimens.

Finally, despite the promise of improved pharmacokinetics, including increased absorption and distribution, nanoparticle-based formulations come with their own set of difficulties relating to production, stability, potential toxicity, and unpredictable results due to variability in absorption. Contrarily, while well established, conventional statins have drawbacks in bioavailability, adverse effects, potential interactions, and frequency of administration, necessitating careful monitoring and possible adjustments to co-administered medications. To optimise dyslipidaemia treatment strategies, it is critical to continually weigh these limitations against the therapeutic advantages as research advances.

## Clinical implications

The clinical implications of the application of the nanoparticle-based statin medication are quite promising. As shown, statin-encased nanocarrier medications have demonstrated favourable pharmacokinetic properties compared to their conventional counterparts. While more human trials are required to further explore the clinical potential of these formulations, it can be proposed that these drug delivery systems can be utilised to achieve the desired therapeutic effects at substantially lower doses. This would consequently result in a dramatic decrease in drug-related adverse effects that are unfortunately associated with statin therapy. A systematic review conducted in 2020 with 120,456 patients analysed the relationship between statin use and adverse events, showing that patients undergoing statin therapy had a higher incidence of self-reporting muscle symptoms, liver dysfunction, renal dysfunction, and eye-related symptoms (Cai et al. 2021). Based on the current literature available, it appears that statin-encased nanocarriers allow a more efficacious and safer option in the treatment of dyslipidaemia with pharmacotherapy. In an open-label dose-escalation clinical trial of a 5-day repeated intramuscular administration of pitavastatin-incorporated poly (lactic-co-glycolic acid) nanoparticles (NK-104-NP) in patients with chronic limb–threatening ischemia (CLTI) conducted in 2022, no cardiovascular or other serious adverse events caused by NK-104-NP were detected during the follow-up period. While more research and human trials are required, there appears to be no reason why nanocarriers cannot achieve desired therapeutic targets at lower doses. Aside from this, the potential for adverse drug reactions caused by statin-encased nanocarriers is likely to arise from the nanocarriers themselves and not the statin medication. This however is a substantial concern, and further study into the adverse effects of nanocarrier drug delivery systems is required.

## Conclusions and future prospects

The promises nanoparticle drug delivery systems made have been largely realised in statin medication. New nanoparticle statin medication outperforms conventional statins in nearly every pharmacokinetic category but, most importantly, provides significant improvements to conventional statins’ poor oral bioavailability. However, what was not anticipated were the unique and complex challenges that arise in conjunction with new technology. These are issues of resources, logistics, production, storage, and transportation. Presently, the universal application of nanoparticle statin medication has yet to be feasible. However, the data presented in this review supports these pragmatic observations. The prospect of a complete replacement of conventional statin medication is likely as nanoparticle technology becomes more widely accepted and accessible. Furthermore, the likelihood of expanding statin nanoparticle application to treat other diseases, such as cancer and increased prevalence in managing cardiovascular disease, is also promising. The development of statin medication drug delivery technology is significant for the future of global healthcare, as increased efficacy and diversification of therapeutic applications are areas of vast potential for global populations and the industry.

## Data Availability

No datasets were generated or analysed during the current study.
